# New Series of the Medical Examiner

**Published:** 1845-01

**Authors:** 


### THE MEDICAL EXAMINER.

PHILADELPHIA, JANUARY, 1845.

The present number of the Examiner is the commencement, not only of a new year and a new volume, but of a *new series*, in which the past character of the work is to be materially changed. Instead of appearing every alternate Saturday, henceforth it will be published monthly—and in place of twenty-four pages of matter each month, it will embrace seventy-two. The advantages, which we expect to derive from this change, were briefly stated in our last number, and need not be repeated. For the details in reference to publication, and the general scope of the work, we must again refer our readers to the advertisement of the Publishers on the cover, merely premising a few words in relation to what more immediately concerns ourself.

In entering upon the task before us, we feel not as one without experience. The past year has sufficed to show us some of the difficulties that beset an editor's course—the labour it involves, the responsibility it brings, and the groundless suspicions and censure it provokes: nevertheless, it is not without utility and some pleasures to recommend it.

The original communications published during the last year, with few exceptions, have been extensively copied into other journals, in Europe and America; which has convinced us that they were valuable, and added much to the interest of the journal. This department, we expect, under our new arrangement, will be materially increased. Of selected matter, from our extensive exchanges, foreign and domestic, we shall always have an abundance at command, and from that abundance we shall endeavour to choose wisely. Greater space will be given to bibliographical notices; and it is here, doubtless, that we shall encounter our chief annoyances. Objects appear differently when viewed from different points; and it rarely happens that readers and reviewers contemplate a book with the same delight as he who has written it. The author, of course, sees no defects in his performance, or he would correct them before committing it to the scrutiny of unbiassed read ers, and therefore he is unprepared to believe in the existence of blemishes, where his partial eyes have seen nothing but what is rich in thought and beautiful in expression. But reviewers as well as authors may and often do err, without sinister motives. Conscious of this, we shall not willingly censure where we can find reasons to approve, and when charged with wrong shall plead not infallibility but an honest purpose. Still, truth and science have claims upon us greater than friendship for authors and publishers, or regard for our own interest; and hence that justice, which would compel us to accord what is due to labour, genius or learning, will require of us to withhold praise where it is not merited. In pursuing the course which we have indicated, it is our fixed determination not to be drawn into long or bootless discussions, nor, on any account, into such as are personal. Early conviction, and long established habit, will sustain our resolves on this point.

